# Genomic insights into the population structure, antimicrobial resistance, and virulence of *Brachyspira hyodysenteriae* from diverse geographical regions

**DOI:** 10.1128/spectrum.03386-24

**Published:** 2025-04-24

**Authors:** Maria Hakimi, Fangshu Ye, Anugrah Saxena, Xiao Hu, Huigang Shen, Paarthiphan Elankumaran, Chloe C. Stinman, Nubia Macedo, Orhan Sahin, Eric R. Burrough, Ganwu Li

**Affiliations:** 1 Department of Veterinary Microbiology and Preventive Medicine, Iowa State University, Ames, Iowa, USA; 2 Department of Statistics, Iowa State University, Ames, Iowa, USA; 3 Veterinary Diagnostic Laboratory, Iowa State University, Ames, Iowa, USA; 4 Department of Veterinary Diagnostic & Production Animal Medicine, Iowa State University, Ames, Iowa, USA; Centre de Biologie Integrative, Toulouse, France

**Keywords:** *Brachyspira hyodysenteriae*, population structure, whole genome sequencing, swine dysentery, phylogenomic analysis, MLST, AMR, virulence

## Abstract

Swine dysentery, caused by the anaerobic spirochete *Brachyspira hyodysenteriae,* leads to mucohemorrhagic diarrhea in grower-finisher pigs, impacting swine production. Knowledge regarding its genomic epidemiology is limited. We performed a whole-genome sequence analysis for 251 *B. hyodysenteriae* genomes from 10 countries, including 117 isolates sequenced in this study. Phylogenomic analysis based on core-genome single nucleotide polymorphisms (SNPs) revealed nine lineages, with L7 (72 isolates, 28.69%), L9 (67 isolates, 26.69%), and L2 (53 isolates, 21.12%) predominating. Geographical clustering was observed with distinct lineage distributions. Multilocus sequence typing identified 69 sequence types (STs), including 20 novel STs across 251 genomes. Association between specific lineages, STs, and geographical regions was evident, highlighting evolutionary and regional patterns. The pan-genome analysis identified 5,231 genes, categorized into core (1,648), accessory (2,619), and unique (964) components. Functional annotation linked core genes to essential cellular processes, while accessory and unique genes were enriched in genetic variability, defense mechanisms, and secondary metabolism. The pan-genome exhibited a high proportion of hypothetical genes, necessitating further functional characterization. Antimicrobial resistance (AMR) screening detected the *tva*(A) and *lnu*(C) genes associated with tiamulin and lincomycin resistance, respectively, in specific lineages and STs. Virulence factor analysis identified genes linked to hemolysin production, iron uptake, and survival in host environments in most isolates, with a subset of genes demonstrating lineage-specific associations that are further linked to pathogenic potential. This comprehensive genomic epidemiological analysis elucidates the genetic diversity, antimicrobial resistance, and virulence of *B. hyodysenteriae* globally, enhancing understanding of its epidemiology and guiding interventions to mitigate swine dysentery.

**IMPORTANCE:**

*Brachyspira hyodysenteriae*, the primary causative agent of swine dysentery, remains a less-studied pathogen than other bacterial species that impact animal health. This study uses whole-genome sequencing and advanced phylogenomic approaches to reveal the genetic diversity and geographical distribution of *B. hyodysenteriae* isolates, focusing on U.S. populations. The identification of nine distinct phylogenetic lineages and associated sublineages highlights the pathogen’s complex population structure and regional variation. Importantly, the study detects AMR genes, including *tva*(A) and *lnu*(C), linked to tiamulin and lincomycin resistance, that may pose significant challenges to disease management. The analysis also identifies virulence-associated genes, shedding light on molecular mechanisms underlying pathogenicity. By combining core-genome SNP phylogenies with multilocus sequence typing and accessory genome insights, this work provides a robust framework for a better understanding of *B. hyodysenteriae* evolution. Overall, these findings underscore the importance of genomic surveillance in informing control strategies and improving swine health worldwide.

## INTRODUCTION

Swine dysentery (SD) is a severe mucohemorrhagic diarrheal disease targeting the large intestine of mainly grower-finisher pigs, while it is less frequently observed in weaner pigs (1). The disease has been reported in swine-rearing countries worldwide and contributes to a significant economic burden globally (2–7). This economic cost is primarily due to poor feed conversion, slower growth rates, and increased mortality (1, 8). The primary etiological agent of SD is *Brachyspira hyodysenteriae*, a gram-negative, fastidious, obligate anaerobic spirochete that colonizes the mucosal lining of the large intestine of pigs (1). In the late 1990s, clinical SD was uncommon in the United States, which slowed down research in North America (8). However, it persisted in other swine-rearing countries globally (9–11). SD reemerged as a clinical concern in the United States and Canada in the late 2000s, but the underlying reasons and mechanisms are not precisely known (3, 11).

The strong hemolytic phenotype of *Brachyspira* spp. is regarded as a primary virulence trait, and hemolysin is considered a key contributor to the pathogenesis of SD (12–14). Eight hemolysin-related genes have been identified in *Brachyspira*, including four known hemolysin genes: *tlyA* (BHWA1_00238), *tlyB* (BHWA1_01228), *tlyC* (BHWA1_01427)*,* and *hlyA* (BHWA1_02643) and four additional putative genes (hemolysin [BHWA1_00587], hemolysin activation protein [KU215649], hemolysin III [BHWA1_00446], and hemolysin channel protein [BHWA1_01870]). However, the specific roles of these genes in contributing to strong hemolysis are not fully understood (15). Several other genes have also been associated with virulence in *Brachyspira* spp., including those encoding outer membrane proteins, iron uptake systems, flagellin, and NADH-oxidase (16–18). Additionally, studies have shown that *B. hyodysenteriae* strains missing the 36 kB plasmid or specific genes on the plasmid exhibit a low pathogenetic potential (19–21) though these need to be investigated further. Traditionally, the treatment and control of SD have relied heavily on antimicrobial use, as there are no commercially available vaccines to prevent the disease (10). Unfortunately, the emergence of antimicrobial resistance (AMR), including multidrug resistance to commonly used antimicrobials, has severely compromised treatment efficacy (3, 22–24). Moreover, new strains of *B. hyodysenteriae* with low virulence potential have emerged in pigs across Europe and Australia (25). This highlights the need for comprehensive studies on *B. hyodysenteriae* to better understand its epidemiology, virulence, and antibiotic resistance (8).

Recent studies using whole-genome sequencing (WGS) have enhanced our understanding of the *B. hyodysenteriae* genome (6, 24, 26). Additionally, genome sequencing efforts, such as the sequencing of *B. hyodysenteriae* strain WA1, have provided important insights into the genetic basis of virulence and adaptation to the porcine large intestine environment (20). In summary, the findings from the WA1 strains highlight the complex interaction of genetic diversity, virulence factors, and AMR, which emphasizes the importance of genome sequencing studies in revealing the molecular mechanisms underlying the pathogenicity and adaptability of *B. hyodysenteriae* (20).

Despite advancements in whole-genome sequencing, significant gaps remain in our understanding of *B. hyodysenteriae*. Previous studies have often focused on limited sample sizes, restricted geographical regions, or narrow aspects of pathogen biology, such as antimicrobial resistance and virulence (6, 24–28). Comprehensive genomic analyses of *B. hyodysenteriae* are still sparse. This has left critical questions about the pathogen’s diversity, evolution, and global spread largely unanswered (6, 27, 28). These are crucial for understanding the full spectrum of genetic diversity and the molecular epidemiology of *B. hyodysenteriae*. This study addresses these gaps by performing a whole-genome sequence analysis of *B. hyodysenteriae* genomes from various regions within the United States, different European countries, and Australia.

## MATERIALS AND METHODS

### Bacterial isolates

A total of 117 isolates were obtained from the Iowa State University Veterinary Diagnostic Laboratory (ISU VDL) culture collection. These isolates originated from clinical diagnostic and surveillance submissions to ISU VDL between 2009 and 2023. Moreover, the isolates originated from 17 different swine production systems some of which included multiple sites or farms (Supplementary File 1). Isolates were recovered from 80°C frozen stocks and passaged on trypticase soy agar (TSA; BD Difco) supplemented with 5% bovine blood, followed by anaerobic incubation at 42°C for 48 hours. Species confirmation was performed using MALDI-TOF MS following the manufacturer’s (Bruker) protocols in place at ISU VDL.

In addition to these isolates, 134 publicly available whole-genome assemblies from GenBank were included in this study for comparative analysis (Supplementary File 1). These genomes originated from 10 different countries, predominantly from Belgium (*n* = 80), Switzerland (*n* = 18), the United States (*n* = 17), Germany (*n* = 7), and Australia (*n* = 5), with other countries represented less frequently. This brought the total number of genomes included in the downstream analysis to 251. The 251 genomes also included the reference strains B204 and WA1.

### Whole-genome sequencing and assembly

The 117 *B. hyodysenteriae* isolates were subcultured on fastidious anaerobic agar (FAA; Neogen) supplemented with 5% defibrinated horse blood (Remel, Thermo Fisher) and incubated anaerobically for 3 days at 42°C. MALDI-TOF MS again confirmed the growth from FAA plates as *B. hyodysenteriae*. Genomic DNA extraction and sequence library preparation were conducted as previously described (29, 30). In brief, genomic DNA was extracted from the pellets using MagMAX Pathogen RNA/DNA Kit with KingFisher Flex System (Thermo Fisher Scientific) according to the manufacturer’s instructions. DNA sequencing libraries were prepared using the Nextera XT DNA Library Preparation Kit (Illumina, CA). DNA quantity was assessed by a Qubit fluorometer with Qubit 1× dsDNA HS kit (Life Technologies Carlsbad, CA). The libraries were pooled and sequenced by the Illumina MiSeq sequencer using either MiSeq reagent kit v2 (500 cycles) or MiSeq reagent kit v3 (600 cycles), generating paired-end read of 25 bp following standard Illumina protocols.

Sequencing coverage for the 117 isolates ranged from 42× to 342×, with most genomes achieving an average coverage depth of over 100×. Raw reads were quality-checked using FastQC and MultiQC, and low-quality reads were filtered and trimmed using Trimmomatic 0.39 (31). Adapter sequences were also removed during this process to ensure all reads met a quality threshold of ≥30 Phred score. Trimmed reads were subsequently re-evaluated with FastQC and MultiQC to confirm the removal of low-quality sequences. *De novo* assembly was performed using SPAdes Genome Assembler version 3.15.5-Linux (32) with default settings and the “—careful” option to minimize mismatches and short indels in the final contigs. Quality assessment of assembled contigs was performed using Quast (33), revealing a consistent GC content of approximately 27% and total genome lengths ranging between 2.9 Mbp and 3.1 Mbp. These quality metrics confirmed that the assembled genomes were suitable for downstream analyses. In addition, average nucleotide identity (ANI) analysis on the sequenced genome was performed to assess genetic variability and confirm phylogenetic relationships. Only isolates with ANI ≥95% were included in this study.

### Phylogenomic analysis

Genomic sequence annotations for all 251 *B. hyodysenteriae* genomes were done using Prokka v1.14.6 (34). The annotated core genes were aligned with Panaroo v1.3.4, and single nucleotide polymorphisms (SNPs) from the core genes were extracted using the SNP-sites v2.5.1 (35). The phylogenetic tree based on core-genome SNPs was constructed using IQ-TREE v2.1.4 (36). We used IQ-TREE’s ModelFinder to automatically select the best-fit substitution model, which resulted in GTR + F + ASC + R6 based on the Bayesian Information Criterion. This model includes ascertainment bias correction (+ASC), ensuring appropriate handling of SNP-only alignments. The resulting tree was midpoint rooted using Newick Utilities v1.6 (37). To account for potential overrepresentation of closely related isolates, particularly those from NCBI originating from the same BioProject, we utilized the core genome SNP-based approach, which offers high-resolution strain differentiation. Fastbaps v1.0.8 (38) was employed with the “baps” option for hierarchical Bayesian clustering of genomes on the phylogenetic tree. Multilocus sequence typing (MLST) was conducted using the PubMLST database for *Brachyspira* (https://pubmlst.org/organisms/brachyspira-spp). For *B. hyodysenteriae* genomes obtained from NCBI lacking assigned sequence types (STs), permissions were sought from submitters to submit these genomes to the PubMLST database for allele and ST assignment. For each genome, the allelic profiles and STs were determined by querying the respective assembled FASTA files against the PubMLST database. For genomes with novel alleles or STs, the assembled FASTA files were submitted to the PubMLST database to receive new allelic numbers and ST assignments. These new STs were subsequently deposited into the PubMLST database.

### PAN-genome analysis

In addition to the core gene alignment file, Panaroo also produced various output files that were used for in-depth pan-genome and core genome analysis. The gene sequences from Panaroo output were used to functionally characterize the gene families within the pan-genome. The gene families within the pan-genome were functionally characterized by the Clusters of Orthologous Genes (COG) (39) using eggnog-mapper v2.1.12 (40).

### AMR and virulence gene detection

ABRicate v1.0.1 (https://github.com/tseemann/abricate) (41) was utilized to screen each *B. hyodysenteriae* genome against multiple publicly available and custom databases. The public databases included ResFinder, CARD, MEGARes, ARG-ANNOT, and the NCBI database. The screening process generated outputs for each database, providing a detailed account of the detected resistance genes. In addition, the output files included percentage coverage and percentage nucleotide identity. We set the threshold for minimum coverage to ≥80% and for minimum identity to ≥80%.

For the detection of virulence genes, we used ABRicate to screen for virulence genes, employing both the Virulence Finder Database (42) and a custom database tailored to *B. hyodysenteriae*. The custom database contained sequences for a total of 19 genes associated with various aspects of bacterial function and pathogenicity, including those related to hemolysin production, iron uptake, and four genes previously associated with virulence (19), which were originally described as being located on the 36 kb plasmid of *B. hyodysenteriae* strain WA1 (20). (Supplementary File 2). The output files generated by ABRicate included the same type of data as for the resistance genes. We set the threshold for minimum coverage at ≥80% and minimum identity at ≥80% for the detection of virulence genes.

## RESULTS

### Phylogenomic analysis and geographical distribution

Whole-genome sequencing was conducted on 117 *B. hyodysenteriae* isolates from the United States using the Illumina MiSeq platform. These isolates originated from 7 different states and 17 swine production systems within the United States. They were obtained from both clinical cases and surveillance samples, all collected from pigs except for one from a rodent, providing a representative snapshot of the pathogen’s genetic diversity within the U.S. swine population.

**Fig 1:**
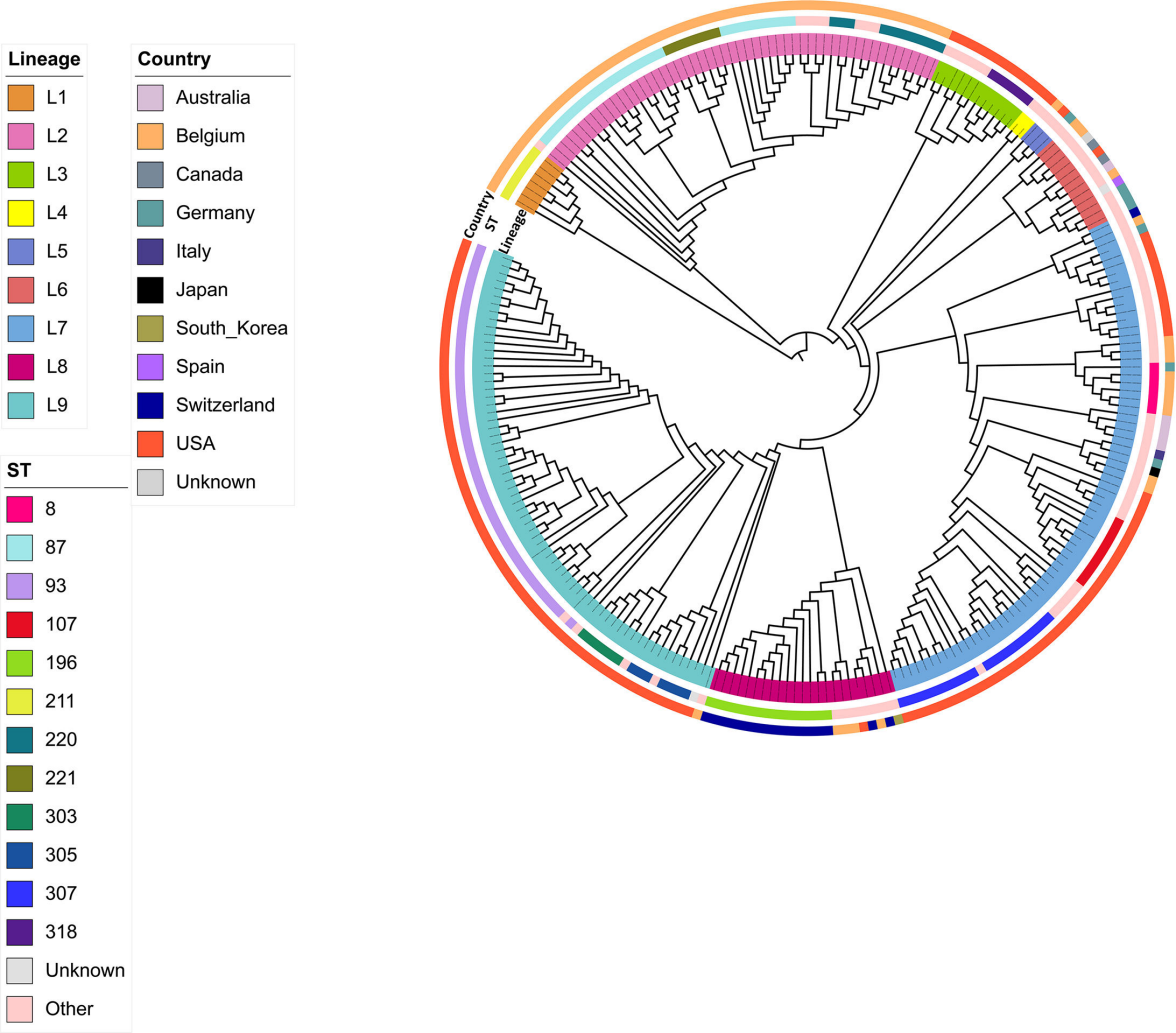
Circular phylogenetic tree based on core-genome SNPs of 251 *B. hyodysenteriae* genomes. The tree was constructed using IQ-TREE v2.1.4 from a multiple alignment of core-genome SNPs generated by Panaroo. The inner ring displays nine distinct lineages defined by the fastbaps “baps” option, with each lineage represented by a unique color. The middle ring displays sequence types (STs), with predominant STs shown in distinct colors, while STs detected five or fewer times are labeled as “other.” The outermost ring shows the countries of origin, with distinct colors representing each country. The legends on the left side of the figure correspond to these rings, providing a visual guide for interpreting the lineages, STs, and countries of origin.

In addition, publicly available sequencing data of 134 *B. hyodysenteriae* genomes originating from 10 different countries such as Australia (5), Belgium (80), Canada (2), Germany (7), Italy (1), Japan (1), South Korea (1), Spain (1), Switzerland (18), the United States (17), and with a single genome with unknown geographical origin, all of which were publicly available from the NCBI database, were also included in this study. The core-genome SNPs-based phylogenetic tree for 251 *B. hyodysenteriae* genomes was constructed by IQ-TREE v2.1.4 (36) from a multiple alignment of core-genome SNPs produced by Panaroo v1.3.4 (43). Subsequently, fastbaps, a fast genetic clustering algorithm (38), was employed to analyze the pre-existing core-genome SNPs-based phylogenetic tree. This approach separated the 251 genomes into nine distinct phylogenetic lineages (lineages L1-L9) (Fig. 1).

Further examination of the composition of each lineage revealed varying sizes and the presence of sublineages within specific lineages. For instance, Lineage L7 was the largest, comprising 72 isolates (28.69%), suggesting a substantial representation within the data set. Lineage L9 contained 67 isolates (26.69%), and Lineage L2 had 53 isolates (21.12%). Each of the three lineages contained sublineages, suggesting additional levels of genetic diversity and potential evolutionary divergence within these groups.

A clear geographical distribution of lineages was observed among the *B. hyodysenteriae* isolates across different countries (Fig. 1). In the United States, lineages L9 and L7 were predominant, representing 49.25% (66/134) and 38.06% (51/134) of the U.S. isolates, respectively. In addition, lineages L3 (8.96%, 12/134), L4 (1.49%, 2/134), L5 (0.75%, 1/134), and L6 (0.75%, 1/134) were also present among U.S. isolates, though in smaller numbers.

The single U.S. *B. hyodysenteriae* isolate from a rodent clustered in one of the sublineages within lineage L9. Belgium isolates primarily clustered within lineage L2, comprising 66.23% (53/80) of isolates from this country. However, there were also isolates from Belgium present in several other lineages (L1, L5, L6, L7, L8, L9), accounting for smaller percentages of the total Belgian isolates. Isolates from Switzerland mainly clustered within lineage L8, accounting for 94.4% (17/18) of Swiss isolates, while German isolates showed a more diverse distribution among lineages L5, L6, and L7. Such patterns may suggest potential regional differences in the distribution of *B. hyodysenteriae* genetic lineages within European countries. Finally, Australian isolates fell mainly within L7, representing 80% (4/5) of the Australian *B. hyodysenteriae* isolates. The number of isolates from the remaining countries was low, which may have prevented their classification into a specific lineage.

### Multilocus sequence typing

After querying the PubMLST database for *Brachyspira* species (https://pubmlst.org/organisms/brachyspira-spp) using the assembled FASTA files of *B. hyodysenteriae* genomes both from ISU VDL and NCBI, a total of 69 different sequence types (STs) were identified based on the analysis of the seven house-keeping genes (Supplementary File 1). At the time of this study, approximately 300 STs were recorded in the PubMLST database, indicating that our data set represents a subset of the known global diversity. Among the 69 STs identified, 20 were novel (STs 317–318, 320–337), while the remaining isolates belonged to previously known STs. Of the 20 novel STs, nine contained at least one novel allele. A clear distribution of all STs is illustrated in Fig. 1, showing their relation to lineages and geographical regions. STs detected five or more times are indicated on the tree, while STs detected fewer than five times are labeled as “other” (Fig. 1). The predominant detected STs detected in the data set were ST93 (*n* = 48), followed by ST87 (*n* = 27), ST307 (*n* = 20), ST196 (*n* = 15), ST220 (*n* = 11), ST107 (*n* = 9), ST211 (*n* = 7), ST221 (*n* = 7), ST305 (*n* = 7), ST8 (*n* = 6), ST303 (*n* = 6), and ST318 (*n* = 6).

The analysis revealed that specific STs were associated with distinct lineages within *B. hyodysenteriae*. For instance, ST93 was exclusively found within lineage L9, while ST87 was observed within lineage L2 only. As mentioned earlier, L2, L7, and L9 exhibited considerable genetic diversity, each containing multiple sublineages. This diversity was reflected in the distribution of the STs within these lineages, with certain STs being specifically associated with particular sublineages. For example, ST307 was observed across two sublineages of the lineage L7, while ST196 was found within lineage L8. Additionally, ST220 was linked to one of the sublineages of L2, and ST107 was found within a sublineage of L7. Similarly, ST211 was associated with lineage L1, and ST221 was observed within one of the sublineages of the L2. Furthermore, ST305 was present in one of the sublineages of L9, whereas ST8 was confined to one of the sublineages of L7. Finally, ST303 was identified within a sublineage of L9, while ST318 was observed in L3 (Fig. 1; Supplementary File 1).

The geographical distribution of these STs highlighted interesting patterns, revealing associations between certain STs and specific geographical regions (Supplementary File 1). The majority of the U.S. isolates belonged to STs ST93 35.82% (48/134), ST307 14.93% (20/134), ST107 6.72% (9/134), ST305 5.22% (7/134), ST303 4.48% (6/134), and ST318 4.48% (6/134). Similarly, in Belgium, the predominant STs were ST87 33.75% (27/80), ST220 13.75% (11/80), ST211 8.75% (7/80), and ST221 8.75% (7/80). ST196 was the predominant ST in Switzerland, constituting 83.33% (15/18) of Swiss *B. hyodysenteriae* isolates.

In most instances, specific STs were unique to individual countries; however, ST8 was identified in both Belgium and Germany, indicating some shared genetic similarity between *B. hyodysenteriae* isolates from these countries. A detailed overview of STs, including their geographical distribution, is provided in Supplementary File 1. Furthermore, certain STs, such as ST87, ST307, and ST305, were split into distinct sublineages within their respective lineages (L2, L7, and L9, respectively). While MLST analysis is commonly used for bacterial strain differentiation, our study demonstrates that core genome SNP-based analysis offers a much higher level of resolution, enabling us to capture finer genetic variations within *B. hyodysenteriae*.

### Pan-genome analysis

**Fig 2:**
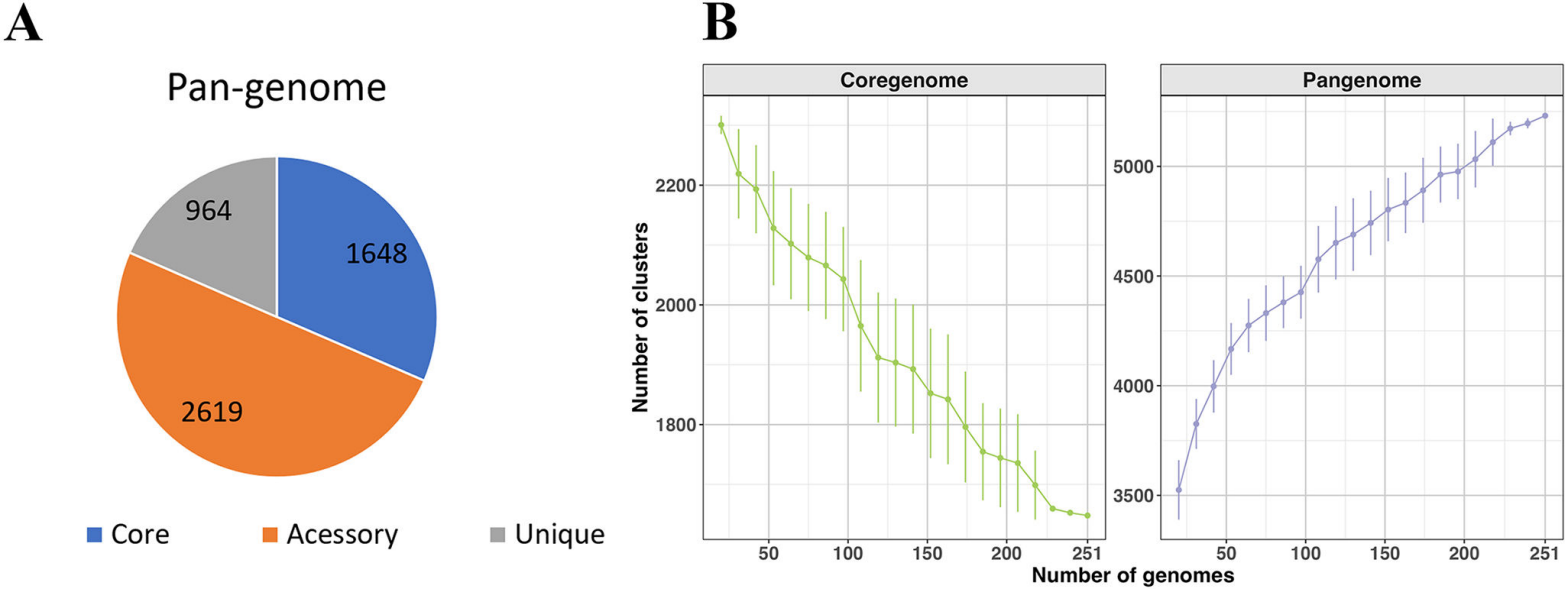
Features of the *B. hyodysenteriae* pan-genome across 251 genomes. (**A**) Pan-genome composition. The pie chart displays the distribution of the 5,231 genes, categorized into core genes (present in all genomes), accessory genes (shared by two or more genomes), and unique genes (specific to a single genome). (**B**) Pan-genome and core genome curves. The curves show the trends observed as more genomes are added to the analysis: a decrease in the number of core genes and an increase in the total number of genes in the pan-genome but closely seem to have reached a plateau. The error bars represent confidence intervals, which decrease as more genomes are included, reflecting reduced variability in estimates.

The pan-genome of *B. hyodysenteriae* was investigated to determine its genetic components. Based on the gene presence-absence matrix provided by Panaroo (Fig. 2A), the pangenome of *B. hyodysenteriae* consisted of 5,231 genes across the 251 genomes analyzed. These genes were categorized into 1,648 core genes (present in 100% of genomes), 2,619 accessory genes (shared by two or more genomes), and 964 unique genes (specific to a single genome). In addition, as shown in Fig. 2B, there was a decrease in the number of core genes with the addition of more genomes to the analysis. Furthermore, the total number of genes in the pan-genome of *B. hyodysenteriae* continued to increase with each additional genome while showing signs of nearing a plateau.

**Fig 3:**
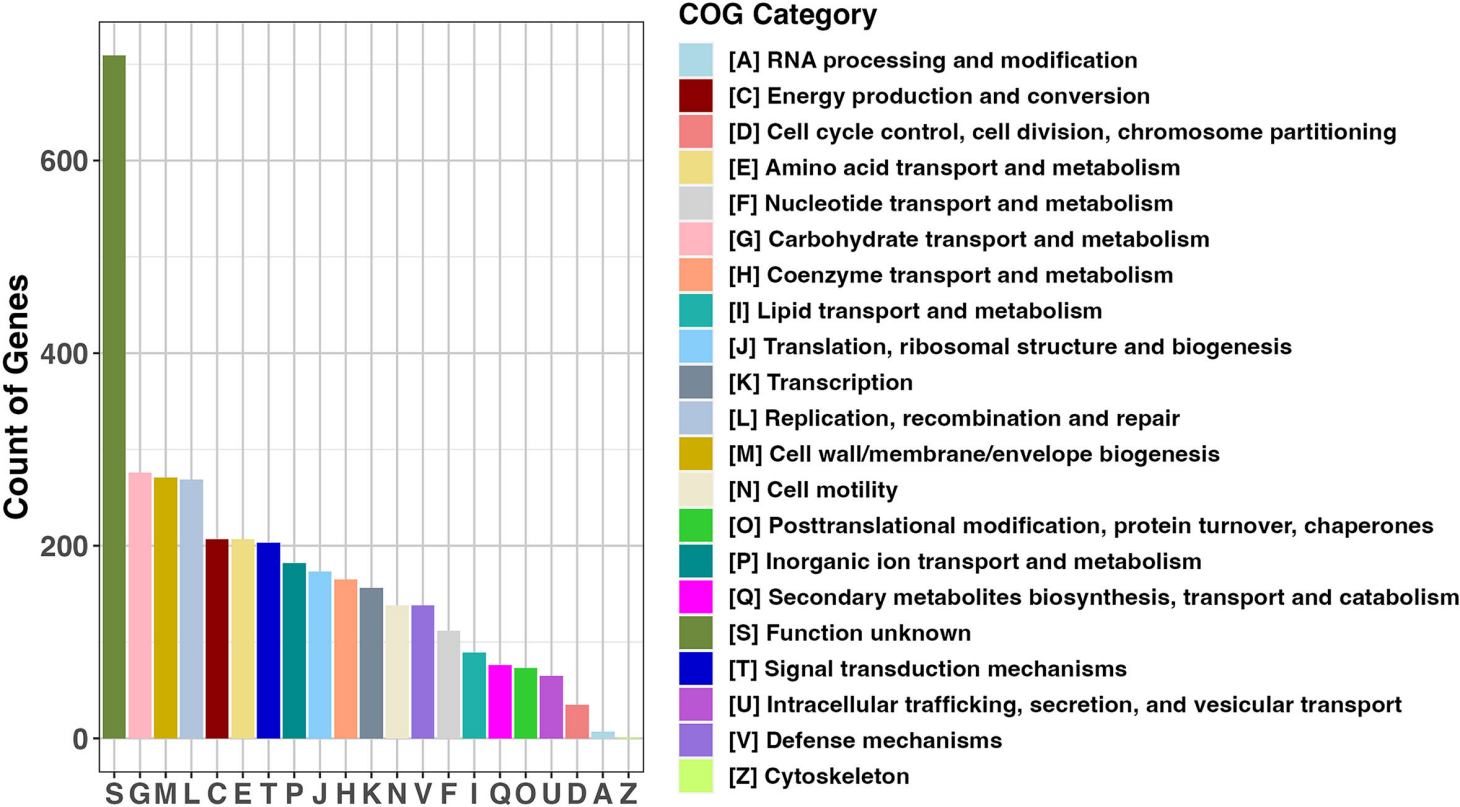
The distribution of Clusters of Orthologous Groups (COG) 21 functional categories in the *B. hyodysenteriae* pan-genome annotated by eggNOG-mapper. Each COG category is represented by a letter and depicted in different colors on the bar graph. The *x*-axis lists the COG functional categories, while the *y*-axis displays the number of genes assigned to each category.

The Panaroo gene presence-absence matrix also provided an annotation description for all 5,231 genes in the pan-genome, revealing that a majority (67.2%) were predicted to encode hypothetical proteins. To achieve a more reliable functional characterization of the gene families identified in the pan-genome, we used the eggnog-mapper software (v2.1.12) (40). The predicted protein sequences generated were annotated using eggnog-mapper to assign orthologous groups and functional categories to the genes. This approach allowed us to categorize each gene into a COG (39) category and provide a functional description using the extensive and curated resources of the eggNOG database (40). Out of the 5,231 genes identified in the pan-genome, only 3,333 of them were assigned to 21 COG functional categories (Fig. 3). In cases where genes were assigned to multiple COG functional categories, each category was counted separately. For example, a gene with a COG designation of “GM” was included in both the “G” and “M” categories. We observed that the gene families assigned to “S: Function unknown” were the most abundant. The next most common categories, albeit in considerably lower frequencies, were “G: Carbohydrate transport and metabolism,” “M: Cell wall/membrane/envelope biogenesis,” and “L: Replication, recombination and repair.” These three categories (G, M, L), while still among the more prominent, were not substantially higher than many of the other COG categories (Fig. 3).

**Fig 4:**
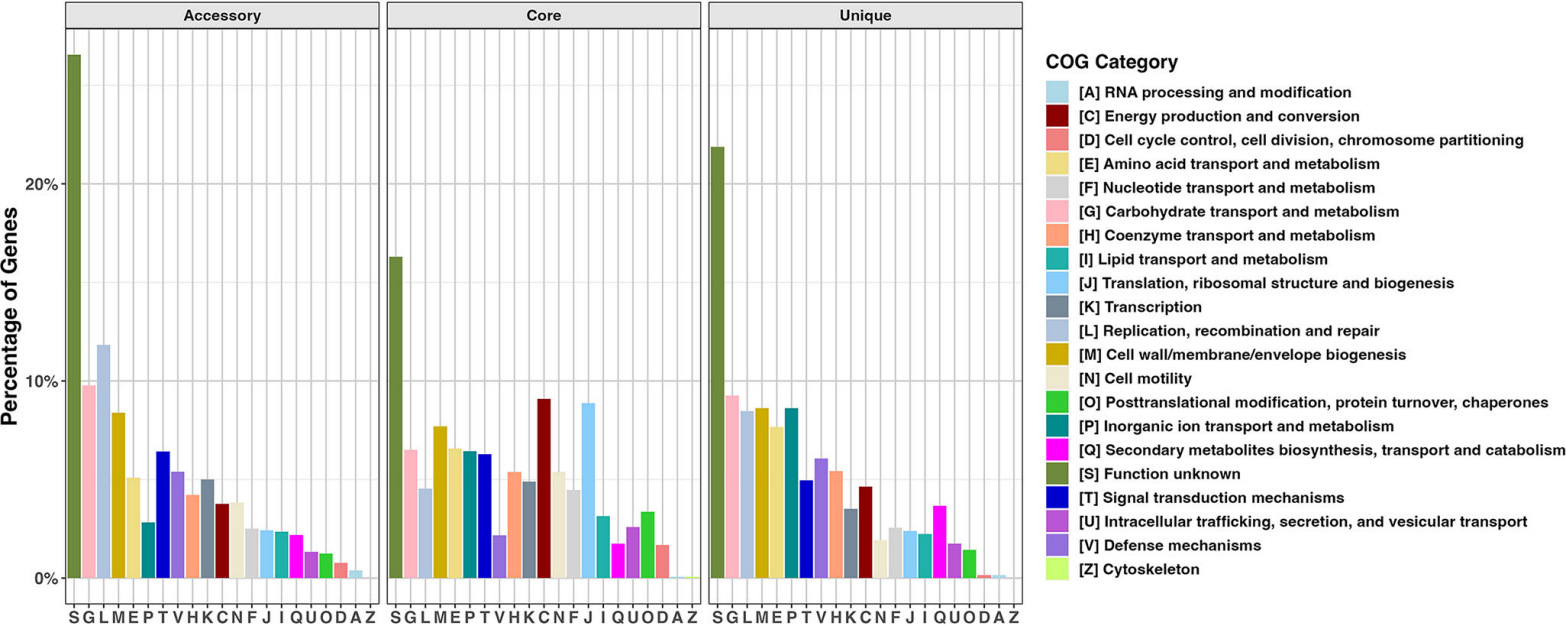
Distribution of COG functional categories across core, accessory, and unique genes in the *B. hyodysenteriae* pan-genome. The *x*-axis shows the COG functional categories. The *y*-axis displays the percentage of genes assigned to each COG category within the core, accessory, and unique gene types.

To better understand the functional composition of the *B. hyodysenteriae* pan-genome, we analyzed the distribution of COG categories across core, accessory, and unique genes (Fig. 4). The core genome was predominantly associated with essential cellular functions such as energy production (C), translation (J), and cell wall/membrane biogenesis (M). In contrast, the accessory and unique genomes contained a higher portion of genes involved in replication, recombination, and repair (L) and defense mechanisms (V), which may contribute to genetic variability. The unique genome showed a slight enrichment in genes linked to secondary metabolism (Q) and inorganic ion transport (P), which may influence niche adaptation. Notably, the “S: Function unknown” category remained the most abundant across all three gene types, underscoring the need for further characterization of these hypothetical proteins.

### AMR gene detection

**Fig 5:**
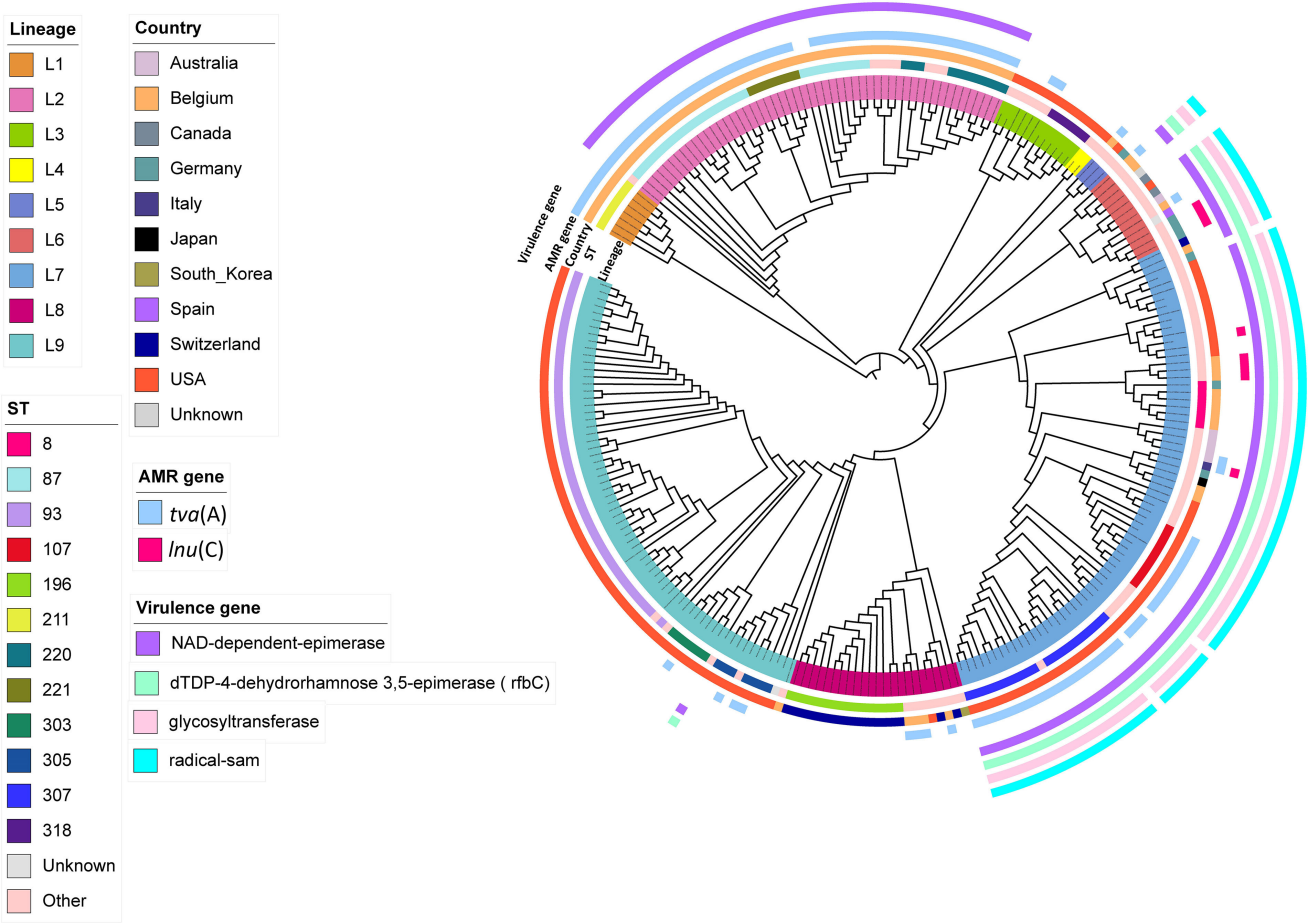
Circular phylogenetic tree based on core-genome SNPs of 251 *Brachyspira hyodysenteriae* isolates. The inner ring displays the nine distinct lineages defined by the Fastbaps “baps” option. The second ring shows the sequence types (STs). The third ring indicates the geographical origin of the isolates. The next two rings highlight the presence of AMR genes *tva*(A) and *lnu*(C). The four outermost rings represent the presence of virulence-associated genes. The legends on the left side of the figure correspond to these rings, providing a visual guide for interpreting the lineages, STs, countries of origin, AMR genes, and virulence genes.

To identify antibiotic resistance genes, we screened all 251 *B. hyodysenteriae* genome sequences using ABRicate software, which accesses multiple antimicrobial resistance gene databases. The screening consistently identified only two genes, *tva*(A), associated with tiamulin resistance (24), and *lnu*(C), associated with lincomycin resistance (44), across the CARD, MEGARes, NCBI, and ResFinder databases. These genes were detected in the same set of isolates (*n* = 113) across all four databases, indicating consistency, while ARGNNOT only detected the *lnu*(C) in eight isolates. For the final analysis and reporting, we relied on the CARD. The detection thresholds were set at ≥80% for both minimum identity and coverage. The genes detected consistently exceeded these thresholds, with coverage ranging from 99.8% to 100% and percent identity reaching a minimum of 93.81%, indicating strong alignment and high confidence in the detection. The distribution of these genes is illustrated in Fig. 5, with detailed information provided in Supplementary File 3.

The *tva*(A) gene was found in 42.23% (106 of 251) of the total isolates, while the *lnu*(C) gene was detected in 3.19% (8 of 251) of isolates. The *tva*(A) gene was notably associated with specific lineages and geographical regions within *B. hyodysenteriae* strains.

The *tva*(A) gene was particularly prevalent in L7 and L2 two lineages. It was detected in 48.61% (35/72) of L7 and was found in nearly all isolates of L2, with a frequency of 96.23% (51/53). In addition, the *tva*(A) gene was present in all isolates of lineage L1. The gene was less common in the remaining lineages (L3, L5, L6, L8, and L9). The *tva*(A) gene was detected in 16.67% (2/12) of L3 isolates, 33.33% (1/3) of L5 isolates, 25% (3/12) of L6 isolates, 17.39% (4/23) of L8 isolates, and 5.97% (4/67) of L9 isolates. Given its association with specific lineages, it is not surprising that the *tva*(A) gene also shows a distinct geographical distribution within *B. hyodysenteriae* strains. In Belgium isolates, which comprised 31.87% (80/251) of the total study isolates, the gene was detected in 81.25% (65/80). In U.S. isolates, which accounted for 53.39% (134/251) of the total study isolates, the *tva*(A) gene was detected in 29.10% (39/134). We also observed an association between the presence of the *tva*(A) gene and specific sequence types (STs). Particularly, the gene was found at a higher prevalence in STs, which represented significant portions of the total isolates. For instance, the *tva*(A) gene was prevalent in ST87, ST307, ST220, ST107, ST211, and ST221. These associations are detailed in Supplementary File 3, which provides a comprehensive overview of the prevalence and distribution of the *tva*(A) gene across different STs.

The *lnu*(C) gene was detected in 3.19% (8/251) of the total isolates. It was primarily observed in lineages L6 and L7. Geographically, the *lnu*(C) gene was found in isolates from Belgium and Germany, a single isolate from the United States, and the only isolate from Italy in our study. Additionally, the gene was associated with ST138 and ST132. Details of these findings are summarized in Supplementary File 3.

### Virulence gene identification

For the detection of virulence genes, we initially used ABRicate against the Virulence Finder Database (VFDB). However, VFDB did not identify any virulence genes in *B. hyodysenteriae* genomes, likely because VFDB does not include *Brachyspira* species in its list of organisms. To address this limitation, we utilized ABRicate with a custom database that contained sequences for 19 genes associated with various aspects of bacterial function and pathogenicity in *Brachyspira hyodysenteriae* (Supplementary File 2). The custom database successfully identified the 19 genes in the *B. hyodysenteriae* genomes, though their presence varied among the study isolates.

We applied thresholds of ≥80% for minimum identity and ≥80% for minimum coverage to the ABRicate output. The coverage for the detected genes ranged from 82.27% to 100%, with most showing near-maximum coverage. Similarly, the percent of nucleotide sequence identity ranged from 85% to 100%, with most hits exhibiting 100% identity.

The distribution of all 19 genes screened, including those related to the hemolysin system, iron uptake, and the four virulence-associated genes, is detailed in Supplementary File 2. The analysis revealed variable frequencies of detection among the virulence genes. Hemolysin-associated genes, including *tlyA, tlyB*, *tlyC*, *hlyA*, and the hemolysin II channel protein gene, were detected in 100% of the isolates (12–15, 45). Hemolysin III and hemolysin III activation protein (12–15, 46) were present in 99.6% and 99% of the isolates, respectively. The iron uptake system is considered a key virulence factor for bacterial pathogens because iron is essential for bacterial growth, metabolism, and survival, yet it is scarce in host environments due to tightly regulated sequestration mechanisms (18, 47). The bit (*Brachyspira* iron transport) system (18) possesses at least three lipoproteins (BitA, BitB, and BitC) with homology to iron periplasmic binding proteins, a protein containing ATP-binding motifs (BitD), and two hydrophobic cytoplasmic membrane permeases (BitE and BitF). Genes *bitA*, *bitB*, *bitC*, *bitD*, *bitF*, and *bitE* were detected in 95.2%, 95.6%, 89.2%, 99.6%, 99.2%, and 100%, respectively. In *B. hyodysenteriae*, the *ftnA* gene encodes the ferritin-like protein FtnA, essential for iron storage, metabolism, and survival in the host, potentially enhancing virulence (47, 48). The *ftnA* gene was present in all isolates. NADH oxidase in *B. hyodysenteriae* is a flavin adenine dinucleotide-dependent enzyme that reduces oxygen to water, facilitating *Brachyspira* protection from oxygen exposure and NADH regeneration for survival and virulence in the swine intestinal tract (16, 49). The NADH-oxidase gene was detected in 100% of the isolates.

A previous study has shown that the absence of specific genes—namely those encoding a predicted radical S-adenosylmethionine domain protein, a glycosyl transferase group 1-like protein, a NAD-dependent epimerase, and a dTDP-4-dehydrorhamnose 3,5epimerase, is associated with a reduced pathogenic potential (19). The detection of these four virulence-associated genes, as shown in Fig. 5, varied across the 251 studied genomes. The NAD-dependent epimerase gene was detected in 53.8% of the isolates, while dTDP-4-dehydrorhamnose 3,5-epimerase (*rfbC*) was found in 33.1%. Additionally, the glycosyltransferase group 1-like protein and the radical SAM domain protein were each detected in 31.5% of the isolates.

Interestingly, the distribution of these four putative virulence-associated genes is closely linked to specific phylogenetic lineages and geographical regions, as illustrated in Fig. 5. The NAD-dependent epimerase gene, in particular, exhibited varying levels of detection across lineages and geographical regions. It was detected in 100% of isolates within lineage L2, which is exclusively associated with Belgium. In lineage L6, although it includes isolates from multiple countries, the gene was predominantly found in German and Belgian isolates, representing 77% of the total isolates within this lineage. For lineage L7, predominantly associated with the United States, this gene was found in 97% of isolates. Additionally, only one isolate from lineage L9, also associated with the United States and originating from a rodent host, tested positive for this gene.

The dTDP-4-dehydrorhamnose 3,5-epimerase (*rfbC*) gene exhibited a similar pattern, detected in 100% of L7 isolates, 77% of L6 isolates (mainly from Germany and Belgium), and the single rodent isolate from lineage L9. Similarly, the glycosyltransferase group 1-like protein was detected in 95.8% of L7 isolates and 77% of L6 isolates, following the same lineage-specific distribution. Finally, the radical SAM domain protein mirrored this pattern, appearing in 95.8% of L7 isolates and 77% of L6 isolates from Germany and Belgium. The consistent distribution across lineages emphasizes the geographic and lineage-specific associations of these virulence-associated genes. Moreover, the known virulent reference strains B204 and WA1, both of which are clustered within lineage L7, harbored all four of these putative virulence-associated genes.

## DISCUSSION

Advancements in WGS have made it possible to gain deeper insights into *B. hyodysenteriae* at the genomic level although prior studies have varied widely in approach and scope. For instance, WGS analysis of isolates from England and Wales revealed genetic similarities to European strains and identified mutations associated with reduced antimicrobial susceptibility (6). Another study focusing on 14 isolates within ST196 uncovered novel prophages and sublineages, highlighting genomic adaptations and evolutionary dynamics (50). Additionally, WGS has been employed to compare weakly and strongly hemolytic isolates, identifying genetic markers unique to a recently emerged sub-clade of weakly hemolytic isolates in Europe (25). Furthermore, WGS has facilitated the discovery of critical antimicrobial resistance elements, including a novel gene *tva*(A), contributing to pleuromutilin resistance and (24) the transposon-associated *lnu*(C) gene linked to lincosamide resistance (44). This approach has also proven instrumental in predicting multidrug resistance profiles (26). While these studies provide valuable insights, they are often limited by restricted geographical representation, insufficient sample diversity, and a focus on specific aspects of the pathogen’s biology, leaving gaps in our broader understanding of *B. hyodysenteriae* on a global scale. Our study addressed these critical gaps by utilizing WGS to analyze 117 *B. hyodysenteriae* isolates from diverse regions across the United States, complemented by 134 publicly available genomes. This comprehensive data set of 251 isolates spans 10 different swine-rearing countries. By combining phylogenomic and pan-genome analyses, along with MLST, our study elucidates the population structure, geographical distribution, and genetic diversity of *B. hyodysenteriae* on a global scale. Additionally, our research examined the prevalence and distribution of antimicrobial resistance genes, virulence-associated genes, and putative virulence genes, offering a more comprehensive understanding of their associations across a broader range of isolates.

A clear, geographically specific distribution of phylogenetic lineages was observed, with most isolates from the United States clustering into lineages L7 (38.06%) and L9 (49.25%), while the majority of isolates from Belgium were grouped within lineage L2 (66.23%). Due to the limited number of sequenced isolates from Australia, Canada, Germany, Italy, Japan, South Korea, Spain, and Switzerland, further studies are necessary to determine the predominant lineage(s) in these countries. The geographic clustering of lineages may be attributed to evolutionary adaptations that enhance survival and transmission within particular environments. Regional factors, such as farming practices and biosecurity measures, likely contribute to this distribution. This pattern could also indicate limited intercontinental spread of specific lineages, suggesting that certain lineages adapt to and thrive in localized settings, thereby limiting cross-region dissemination. In addition, isolates of several lineages were present in multiple European countries (Fig. 1). This observation could be linked to frequent regional trades and animal movements within Europe (6).

The MLST analysis identified 69 different sequence types (STs), including 20 novel STs across the analyzed isolates. Previous studies have also reported a genetic diversity among *B. hyodysenteriae* isolates through MLST typing (6, 11, 51, 52). Mapping the STs to the core genome, SNP-based phylogenetic lineages (Fig. 1) revealed close associations between certain STs, particular lineages, and geographical regions, as anticipated. Core genome SNP-based genotyping provides a greater level of discrimination than MLST typing; isolates with the same STs were found within both the same and adjacent core-genome SNP clusters on the phylogenetic tree. For example, the SNP-based analysis showed that STs like ST87, ST307, and ST305 could further split into distinct sublineages (Fig. 1), highlighting a depth of genetic variation that MLST alone might not fully capture. Supporting this, Stubberfield et al. (6) demonstrated that core genome SNP variation within specific STs can reveal significant differences in genetic stability and diversity, underscoring the enhanced discriminatory power of core genome SNP-based analysis (6).

In analyzing the pan-genome of *B. hyodysenteriae*, we identified 1,648 core genes conserved across 251 genomes, emphasizing the fundamental biological processes shared by the bacterium. The variability in accessory genes (2,619 genes across 251 genomes) and unique genes (964 genes) (Fig. 2A) highlights the genetic diversity within the species, suggesting differences in traits such as virulence, antibiotic resistance, and adaptability to specific environments (53). Our findings contrast with those of Black et al. ) (27) and Garcia-Martin et al. (50), who observed a lower number of accessory genes. This difference may be attributed to the smaller number of *B. hyodysenteriae* genomes analyzed in their study or the limited geographical regions represented (27). Despite these differences, in agreement with Garcia-Martin et al. (50), our study also identified a highly conserved core genome (1,648 core genes) within the *B. hyodysenteriae* pan-genome (28). Additionally, as more genomes were analyzed, a decrease in the number of core genome genes and an increase in the total number of genes in the pan-genome were observed (Fig. 2B). By around 251 genomes, the number of core genome genes appeared to have nearly stabilized, while the total gene counts in the pan-genome had closely reached a plateau.

Due to the lack of commercially available vaccines, control and prevention of SD relies solely on antimicrobials (1, 10). Unfortunately, antimicrobial resistance to commonly used antimicrobials (e.g., pleuromutilins, tylosin, and lincomycin) has been reported in swine-rearing countries (3, 22, 51, 54, 55). The *tva*(A) gene encodes an ABC-F transporter linked to reduced susceptibility to pleuromutilins (24). This gene was detected in 42.23% of the isolates, showing significant prevalence, particularly in lineages L2 and L7, where it was found in nearly all isolates. Geographically, *B. hyodysenteriae* isolates from Belgium more often harbored the *tva*(A) gene (81.25%) compared to U.S. isolates (29.10%). The lower prevalence of the *tva*(A) gene in U.S. isolates may be influenced by the historical decline of swine dysentery in the United States during the late 1990s (8), which led to reduced exposure to antimicrobials commonly used for its treatment and control. This decrease in disease occurrence may have contributed to lower selective pressure for the maintenance of tiamulin resistance determinants in the U.S. pig population. In contrast, in regions such as Belgium, where swine dysentery remains more prevalent, the consistent use of pleuromutilins for treatment could have contributed to the higher frequency of the *tva*(A) gene in isolates from this region (10). In addition, our previous study on 52 *B. hyodysenteriae* isolates from the United States revealed that four (4/52) exhibited phenotypic resistance to tiamulin (51). Notably, all four also harbored the *tva*(A) gene identified in this study. However, the *tva*(A) gene was also detected in four isolates that were phenotypically susceptible to tiamulin. This finding is not surprising, as clinical resistance of *Brachyspira* to pleuromutilins typically requires additional factors, such as mutations in ribosomal proteins or the 23S rRNA gene (24). Additionally, it is possible that the *tva*(A) gene in these cases (i.e., phenotypically tiamulin susceptible isolates) may have been mutated or not fully expressed.

The *lnu*(C) gene encodes an enzyme that deactivates lincosamides, contributing to reduced susceptibility to lincomycin (44). In comparison to the *tva*(A) gene, the *lnu*(C) gene was significantly less common, found in only 3.19% (*n* = 8) of the isolates, primarily in lineages L6 and L7. It was also geographically restricted to Belgium, Germany, and one U.S. isolate. For lincomycin, seven isolates (7/52) displayed phenotypic resistance in our previous study (51). However, only one of these resistant isolates harbored the *lnu*(C) gene. This discrepancy indicates the potential presence of alternative or novel mechanisms of resistance to lincomycin, which warrants further investigation.

Our study also examined the distribution of four virulence-associated genes: NAD*-*dependent epimerase, dTDP-4-dehydrorhamnose 3,5-epimerase (*rfbC*), glycosyltransferase group 1-like protein, and radical SAM domain protein, across the identified lineages (19). Each of these genes exhibited lineage-specific distribution patterns. Notably, the genes encoding dTDP-4-dehydrorhamnose 3,5-epimerase (*rfbC*), glycosyltransferase group 1-like protein, and radical SAM domain protein were uniquely associated with isolates from lineages L6 and L7. In contrast, the gene expressing a NAD*-*dependent epimerase was found not only in isolates from lineages L6 and L7 but also in those from lineage L2. Given that these four genes have been reported as predictive markers of pathogenicity in *B. hyodysenteriae*, it would be valuable to investigate whether isolates from lineages L6 and L7 are more virulent than those from lineage L2 and whether isolates from lineage L2 exhibit greater virulence than those from other lineages. Additionally, since both lineages L7 and L9 are dominant lineages in the United States, comparing the virulence of isolates from L7 against those from L9 could provide further insights. To support these investigations, developing genetic manipulation techniques and conducting animal studies will be essential to clarify the pathogenic roles of these genes.

In summary, our study provides significant insights into the genetic diversity and lineage-specific distribution of *B. hyodysenteriae* across 251 genomes, revealing nine distinct lineages with varying genetic heterogeneity. The identification of 69 different STs, including 20 novel ST types, underscores the high genetic diversity within this spirochete. Our analysis of the pan-genome demonstrated a core set of 1,648 conserved genes alongside a large accessory genome. Moreover, the frequent detection of specific virulence-associated genes in lineages L6 and L7 raises important questions about their potential roles in pathogenicity and the evolutionary dynamics of these lineages. Additionally, the observed patterns of antimicrobial resistance, particularly the distribution of the *tva*(A) and *lnu*(C) genes across specific lineages and geographic regions, underscore the need for further investigation into the interplay between resistance mechanisms and lineage evolution.

While our study enhances the genomic understanding of *B. hyodysenteriae*, the data set is largely composed of isolates from the United States and Belgium due to the availability of genomes from NCBI. The limited representation from other swine-rearing countries reflects data availability rather than intentional selection and underrepresented regions could harbor distinct lineages or resistance patterns. Expanding genome sequencing in this area is essential for more comprehensive understanding of the pathogen’s epidemiology and evolution.

## Data Availability

The whole-genome sequencing data generated for 117 U.S. isolates in this study were deposited in the NCBI database (GenBank accession no. PRJNA1228138). SRA accessions and BioSamples for individual sequences are listed in Supplementary File 1. To facilitate reproducibility, all commands and parameters used in the analysis have been documented and made publicly available on GitHub: https://github.com/isu-vdl-ngs/Brachyspira_hyodysenteriae

## SUPPLEMENTAL MATERIAL

Data S1 spectrum.03386-24-s0001.xlsx Metadata.

Data S2 spectrum.03386-24-s0002.xlsx Virulence gene detection.

Data S3 spectrum.03386-24-s0003.xlsx AMR gene detection.

ASM does not own the copyrights to Supplemental Material that may be linked to, or accessed through, an article. The authors have granted ASM a non-exclusive, world-wide license to publish the Supplemental Material files. Please contact the corresponding author directly for reuse.
